# Scoring prognostic factors for pregnancy post-intrauterine insemination in couples with infertility

**DOI:** 10.11604/pamj.2025.50.64.45962

**Published:** 2025-03-03

**Authors:** Geraldo Laurus, Agustinus Agustinus, Cennikon Pakpahan, Vellyana Lie, Maitra Djiang Wen

**Affiliations:** 1 Bocah Indonesia Fertility Centre, Tanggerang, Indonesia; 2 Andrology Study Program, Faculty of Medicine, Universitas Airlangga, Surabaya, Indonesia; 3 Department of Biomedical Science, Faculty of Medicine, Universitas Airlangga, Surabaya, Indonesia; 4 Wahidin Sudiro Husodo General Hospital, Mojokerto, Indonesia

**Keywords:** Intrauterine insemination, prognostic factors, pregnancy, scoring, reproductive health

## Abstract

**Introduction:**

intrauterine insemination (IUI) was selected as a treatment for infertility due to its simple method, affordability, and non-invasive. Intrauterine insemination had pregnancy rates after one cycle of IUI ranging between 8.2% and 15.1%. Candidates for IUI are couples facing specific infertility including cervical factors, ovulatory dysfunction, endometriosis, and ejaculation. Therefore, this study aimed to assess prognostic factors significantly affecting the outcome of IUI in Indonesia before the IUI procedure.

**Methods:**

this study was conducted at Bocah Indonesia Fertility Clinic, Tangerang, and SMART IVF Anna Hospital, Bekasi, using an observational analytic method with a cohort retrospective design. Data were collected from infertile couples subjected to IUI between 2020 and 2024. The primary outcome measured was biochemical pregnancy, defined as a positive serum human chorionic gonadotropin (hCG) test following IUI. Prognostic factors for pregnancy were assessed and identified, while a predictive scoring system was developed using a multivariate analysis.

**Results:**

a total of 443 IUI cycles were evaluated and a pregnancy rate of 13.5% was observed. Multivariate analysis showed that pregnancy rates were influenced by the body mass index (BMI) of the male (<27.5 kg/m^2^), age of the female (<30.5 years old), total progressive sperm count after sperm preparation (>15.81 million), first insemination, anti-mullerian hormone (AMH) level (>3.015 ng/mL), and endometrial thickness (>9.36 mm). A scoring system ranging from 0-9 was developed, with a probability of pregnancy from 1.31% to 55.16%, for scores 0 to 9, respectively.

**Conclusion:**

the scoring system served as a tool designed specifically to evaluate the relationship between prognostic factors and post-IUI pregnancy rates.

## Introduction

Intrauterine Insemination (IUI) was selected as a treatment for infertility due to the simple method, affordability, and non-invasive nature compared to other procedures, namely in vitro fertilization (IVF) [1-3]. Furthermore, this procedure is relatively safe as there are no associated significant complications [4]. Intrauterine insemination (IUI) can cause various factors such as cervical factors, ovulatory dysfunction, endometriosis, immunological causes in women, ejaculation and coital disorders in men, as well as unexplained infertility.

Several studies have shown that pregnancy rates after one cycle of IUI ranged between 8.2% and 15.1% [5-10]. There were several prognostic factors contributing positively to pregnancy rates. Prognostic factors can be divided into pre-cycle, insemination, and post-cycle factors. Pre-cycle prognostic factors in men, such as age [11] total motility, and normal morphology using strict criteria [7]. Pre-cycle prognostic factors from the female aspect were female age [4,5,7,12,13], endometrial thickness [5,14], the number of mature follicles after stimulation [5,7,15], duration of infertility [7,14,16], and the stimulation protocol used for IUI [5,7,12]. Factors during insemination such as obstruction during catheter placement, abdominal cramps, catheter bleeding, and bleeding during insemination can affect pregnancy rates [17]. Measures facilitating catheter placement were observed to have a positive influence [18]. Administration of progesterone preparations as luteal support is a post-cycle factor that can also affect pregnancy rates [19].

Several studies have evaluated prognostic factors for this treatment success. However, none have analyzed the factors for post-IUI pregnancy rates in the Indonesian population. The results of some investigations conducted in the absence of couples with infertility showed a scoring model for prognostic factors of post-IUI pregnancy rates [20,21]. This study aims to evaluate the relationship between prognostic factors and post-IUI pregnancy rates. The result is expected to be a scoring model to assess rates that are specific and individualistic.

## Methods

**Figure 1:**
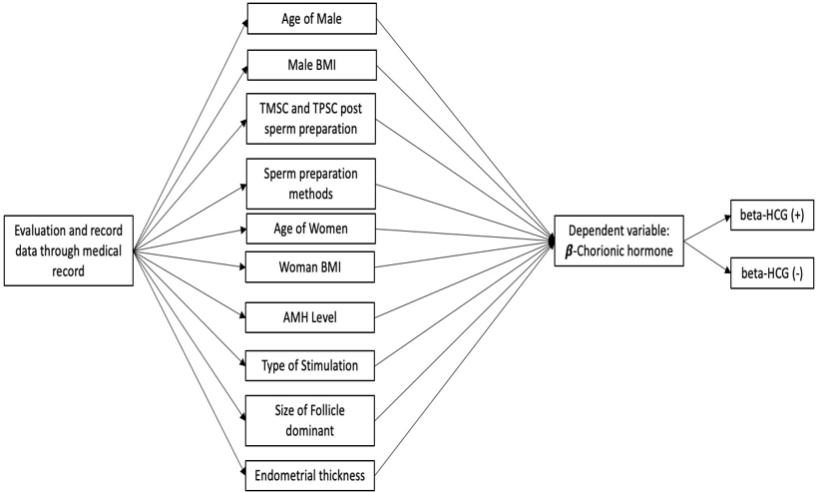
analysis framework in the study

**Study design:** an observational analysis with a retrospective cohort design was adopted to evaluate the relationship between prognostic factors for pregnancy rates in post-IUI patients (Figure 1).

**Setting:** the population of this study was medical records of patients with infertility who sought treatment at Bocah Indonesia Fertility Clinic and SMART IVF Anna Hospital, as well as experienced IUI during the period from 2019 to December 2023.

**Participants:** the participants in this study are medical records of the patients who took IUI Bocah Indonesia Fertility Clinic and SMART IVF Anna Hospital. The criteria comprised data related to IUI in the medical records of couples with infertility who experienced IUI procedures at Bocah Indonesia Fertility Clinic and SMART IVF Anna Hospital Bekasi. We excluded the IUI data containing prognostic factors in incomplete medical records.

**Variables:** there are ten independent variables in this study and one dependent variable. Ten independent variables are the age of the male or female, BMI male or female, TMSC (total motility sperm count) and TPSC (total progressive sperm count) post sperm preparation, sperm preparation method, AMH (anti-mullerian hormone level), type of stimulation, size of the follicle, and endometrial thickness. TMSC refers to multiplying the percentage of motile spermatozoa (rapidly progressive, slowly progressive, and non-progressive) by the volume and concentration before sperm preparation. Total progressive sperm count (TPSC) is the percentage of progressively motile spermatozoa multiplied by the volume and concentration obtained after sperm preparation. Meanwhile, the dependent variable is the beta-chorionic hormone level, with 10 IU as the threshold for pregnancy. This pregnancy was defined as a biochemical pregnancy. We just assessed biochemical pregnancy in this study.

### Data sources and measurement

**Data collection:** the data we extracted from the corresponding medical records included the couple's age, anthropometry, sperm profile, hormone levels, type of female stimulation used, follicle count from ultrasound, and endometrial thickness. All these data were considered as independent variables. We then extracted the patient's pregnancy data by pulling the beta-HCG level data as the dependent variable. All data extraction sheets were designed and used to collect data.

**Sample size:** the sample size is based on the rule of thumb with the formula:


$$10\times n\left(\frac{\text{total of variable}}{p\;\left(\text{propotion}\right)}\right)$$

Eleven variables will be evaluated, and the incidence of subjects with the result of acceptable pregnancy was 30% [22]:


$$\frac{10\times 11}{30\%}=\frac{110}{0.3}=366$$

**Sampling strategy:** the method adopted was total sampling, comprising all samples that met the study criteria and fulfilled the inclusion and exclusion criteria.

**Figure 2:**
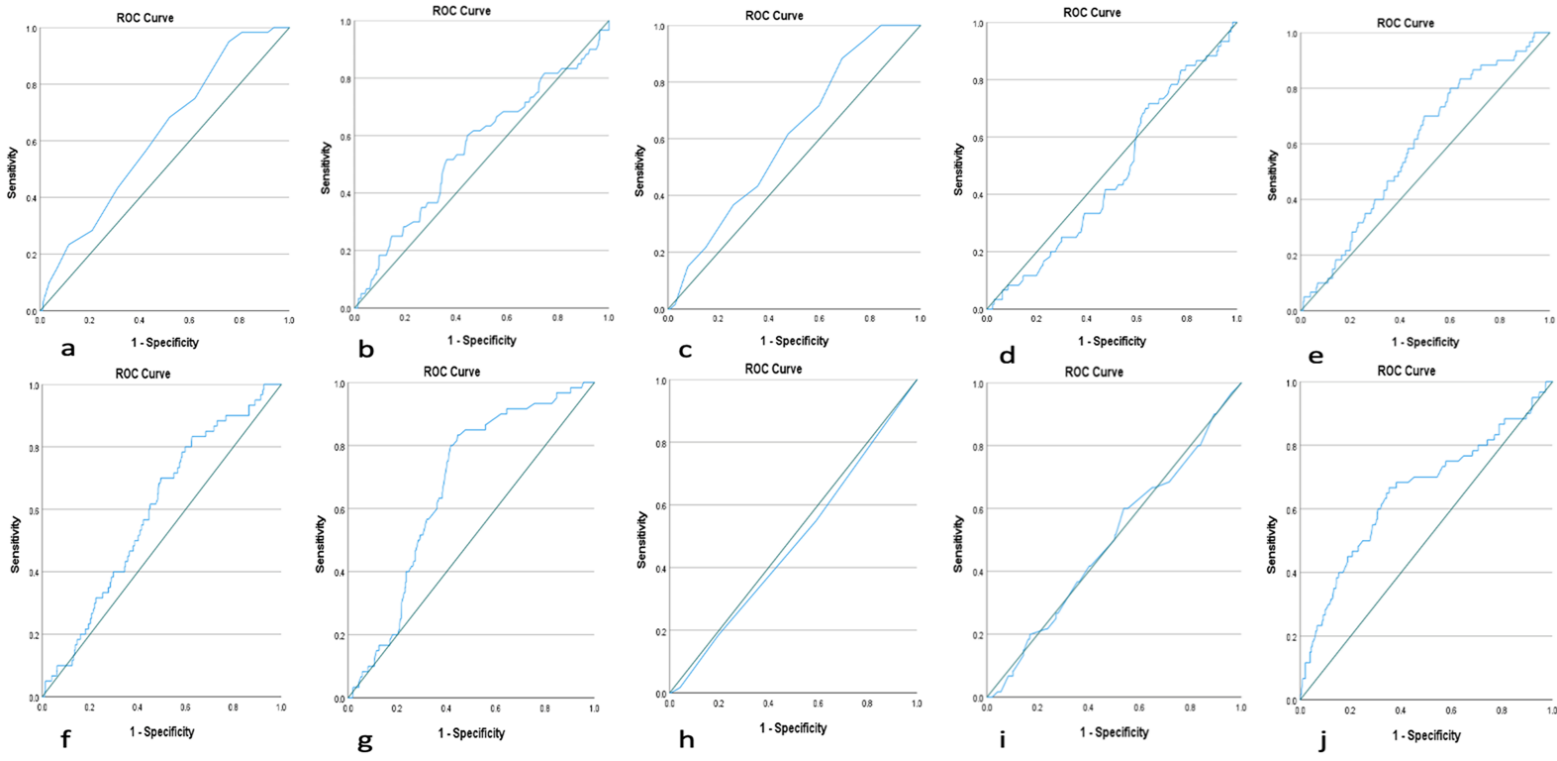
receiver operating characteristic analysis of male and female factors; A) male age; B) male body mass index; C) female age; D) female body mass index; E) total motility sperm count; F) total progressive sperm count; G) female anti-mullerian hormone levels; H) numbers of mature follicles; I) size of mature follicles; J) endometrium thickness

**Data analysis:** this study's data analysis process consisted of six steps using Statistical Package for the Social Sciences (SPSS) version 25.0 (Figure 2). We first conducted a univariate analysis to get the descriptive values or profiles of the participants. Then, conduct a bivariate analysis between independent and dependent variables. Each independent variable that was previously a numeric scale will be determined first with area under the curve (AUC), receiver operating characteristic (ROC), and Youden test analysis. Then, the analysis continued with the logistic regression test by including each bivariate analysis test result with a value of p < 0.25 to calculate the score value of each variable.

**Ethical considerations:** this study had ethical approval and was approved by the University of Airlangga Ethics Committee (339/EC/KEPK/FKUA/2023).

## Results

### Participant descriptive

**Demography and clinical characteristics:** samples comprised medical records of infertile couples who had experienced the IUI procedure at Bocah Indonesia Fertility Clinic, Tangerang, and SMART IVF Anna Pekayon Hospital, Bekasi. The size was 443 couples, of which 60 were fertile (13.5%). The participants’ characteristics are presented in Table 1 which shows several differences in characteristics between pregnant and non-pregnant after the administration of IUI. The mean ages of male partners were 31 and 32.9 years, respectively, indicating a significant difference. In the female partners, the mean ages of the 2 groups were 29.6 and 30.9 years. However, the male and female BMI did not show a significant difference. In terms of TMSC and TPSC post-preparation, couples who successfully conceived with p values 0.021. Furthermore, female factors such as AMH levels and endometrial thickness showed p values < 0,001, and higher pregnancy success. Other factors such as the number of follicles and the size of mature follicles presented no significant differences (p=0.536 and p=0.955). Additionally, differences in sperm preparation methods, number of inseminations, etiology of female infertility, and type of stimulation were not significantly different between the groups (p>0.05).

**Table 1:** participants' characteristics

Characteristics	Total (n=443)	Not pregnant (n=383)	Pregnant (n=60)	P-value
Male age (years)	32.69 ± 4.27	32.95 ± 4.36	31.03 ± 3.16	0.003^c^*
Male BMI (kg/m^2^)	27.48 ± 4.22	27.53 ± 4.05	27.17 ± 5.14	0.162^c^
Female age (years)	30.79 ± 3.51	30.98 ± 3.60	29.58 ± 2.55	0.007^c^*
Female BMI (kg/m^2^)	24.01 ± 4.18	23.96 ± 4.22	24.31 ± 3.92	0.528^c^
TMSC post-preparation (million)	15.67 ± 7.27	15.37 ± 7.32	17.56 ± 6.68	0.021^c^*
Post-preparation TPSC (million)	15.23 ± 7.22	14.94 ± 7.28	17.11 ± 6.58	0.021^c^*
AMH level (ng/mL)	3.16 ± 2.13	3.06 ± 2.15	3.79 ± 1.87	<0.001^c^*
Number of mature follicles	1.82 ± 0.85	1.83 ± 0.86	1.75 ± 0.79	0.536^c^
Diameter of mature follicles (mm)	19.99 ± 2.32	20.00 ± 2.33	19.94 ± 2.22	0.955^c^
Endometrial thickness (mm)	9.09 ± 1.63	8.97 ± 1.55	9.87 ± 1.86	<0.001^c^*
**Preparation method**				0.289^a^
Swim-up	14 (3.2%)	12 (3.1%)	2 (3.3%)	
DGC	384 (86.7%)	328 (85.6%)	56 (93.3%)	
Mixed	35 (7.9%)	33 (30.3%)	2 (3.3%)	
Simple washing	10 (2.3%)	10 (2.6%)	0 (0%)	
**Insemination**				0.127^a^
1	351 (79.2%)	299 (78.1%)	52 (86.7%)	
>1	92 (20.8%)	84 (21.9%)	8 (13.3%)	
**Etiology of female infertility**				0.622^a^
Unknown or not found	133 (30.0%)	118 (30.8%)	15 (25.0%)	
PCOS	61 (13.8%)	51 (13.3%)	10 (16.7%)	
Endometriosis	51 (11.5%)	42 (11.0%)	9 (15.0%)	
Uterine disorders	198 (44.7%)	172 (44.9%)	26 (43.3%)	
**Type of stimulation**				0.057^a^
Mini	127 (28.7%)	116 (30.3%)	11 (18.3%)	
Mild	316 (71.3%)	267 (69.7%)	49 (81.7%)	

Numerical data are presented as mean ± s.d., categorical data are presented as n (%); ^a^Chi-square; bt-test; ^c^Mann Whitney; BMI: body mass index, TMSC: total motility sperm count, TPSC: total progressive sperm count, AMH: anti-mullerian hormone, DGC: density gradient centrifugation, PCOS: polycystic ovary syndrome

### Outcome data

**Receiver operating characteristic analysis determining cutoff variables:** receiver operating characteristic analysis was conducted to determine the scoring of prognostic factors for pregnancy in infertile couples after undergoing IUI. Statistical analysis aimed to evaluate the cutoff values for each variable generated from the AUC curve. Figure 2 showed the results of ROC analysis, AUC, as well as the sensitivity and specificity values of the cutoff for several numeric variables such as the age of male and female, BMI of male and female, TMSC and TPSC post-preparation, AMH levels, number of follicles, size of mature follicles, and endometrial thickness. The results of ROC analysis of all prognostic factors evaluated showed that AMH levels (AUC = 0.667; p<0.001) and endometrial thickness (AUC = 0.651; p<0.001) had the highest AUC values. The sensitivity and specificity values were statistically found slighter than 70% using the regression logistic analysis test. Other factors such as male age, female age, and post-preparation TMSC and TPSC also produced statistically significant ROC (p<0.05).

After analysis ROC and AUC continue to Table 2 shows the bivariate analysis between prognostic factors divided based on cutoff values and pregnancy post-IUI. A significant relationship was observed between AMH levels and endometrial thickness (p<0.001) with pregnancy. Total motility sperm count values were also associated with post-IUI pregnancy in infertile couples (p=0.027). Other variables that show a significant relationship in bivariate analysis were male age, female age, and BMI of males with post-IUI pregnancy (p=0.041, p=0.042 and p=0.036).

**Table 2:** relationship between prognostic factors and pregnancy

Characteristics	Not pregnant (n=383)	pregnant (n=60)	P-value	RR	95%CI
**Male age**			0.041	1.633	1.016-2.626
≥31.5 years	220 (57.4%)	26 (43.3%)			
<31.5 years	163 (42.6%)	34 (56.7%)			
**Male BMI**			0.036	1.655	1.026-2.670
≥27.5 (kg/m^2^)	215 (56.1%)	25 (41.7%)			
<27.5 (kg/m^2^)	168 (43.9%)	35 (58.3%)			
**Female age**			0.042	1.645	1.012-2.675
≥30.5 years	201 (52.5%)	23 (38.3%)			
<30.5 years	182 (47.5%)	37 (61.7%)			
**Female BMI**			0.156	0.711	0.443-1.141
≥24.25 (kg/m^2^)	173 (45.2%)	33 (55.0%)			
<24.25 (kg/m^2^)	210 (54.8%)	27 (45.0%)			
**TMSC post preparation**			0.027	1.701	1.055-2.743
≤16.33 million	218 (56.9%)	25 (41.7%)			
>16.33 million	165 (43.1%)	35 (58.3%)			
**TPSC post-preparation**			0.064	1.560	0.970-2.509
≤15.81 million	215 (56.1%)	26 (43.3%)			
>15.81 million	168 (43.9%)	34 (56.7%)			
**AMH**			<0.001	2.464	1.518-3.999
≤3.015 ng/mL	245 (64.0%)	23 (38.3%)			
>3.015 ng/mL	138 (36.0%)	37 (61.7%)			
**Number of mature follicles**			0.532	0.860	0.537-1.379
<2	156 (40.7%)	27 (45.0%)			
≥2	227 (59.3%)	33 (55.0%)			
**The diameter of a mature follicle**			0.985	0.995	0.622-1.594
≤19.90 mm	191 (49.9%)	30 (50.0%)			
>19.90 mm	192 (50.1%)	30 (50.0%)			
**Endometrial thickness**			<0.001	3.034	1.837-5.012
≤9.36 mm	247 (64.5%)	40 (66.7%)			
>9.36 mm	136 (35.5%)	20 (33.3%)			

BMI: body mass index, TMSC: total motility sperm count, TPSC: total progressive sperm count, AMH: anti-mullerian hormone, CI: confidence interval, RR: relative risk

Multivariate analysis of male and female prognostic factors in pregnancy: multivariate analysis was conducted on all prognostic factor variables with p<0.25 in the bivariate analysis. The analysis adopted the multiple logistic regression analysis procedures and the backward stepwise multivariate binary logistic regression method. The categorical variables included were the age of male and female, BMI of male and female, TPSC post-preparation, AMH levels, endometrial thickness, insemination number, as well as stimulation type. Variables with p<0.1 in the multivariate analysis were considered significant prognostic factors related to post-IUI pregnancy in the scoring system. Table 3 shows the results of the multivariate analysis of prognostic factors for post-IUI pregnancy. The results of the multivariate analysis using the backward stepwise multivariate binary logistic regression method showed that the prognostic scoring model for post-IUI pregnancy included variables such as male BMI, female age, TPSC, AMH levels, endometrial thickness, and first insemination. These variables were identified as significant prognostic factors (p<0.10).

**Table 3:** results of multivariate analysis of prognostic factors for post-intrauterine insemination pregnancy

Factor	B	S.E.	Significance level	Exp (B)	95%CI for EXP (B)		B/SE	(B/SE)/1.651
					Lower	Upper		
Male BMI (<27.5 kg/m2)	0.497	0.301	0.098	1.644	0.912	2.963	1.651	1
Female age (<30.5 years)	0.523	0.303	0.085	1.687	0.931	3.057	1.726	1.05
Post-preparation TPSC (>15.81 million)	0.669	0.305	0.029	1.952	1.073	3.552	1.872	1.13
AMH (>3.015 ng/mL)	1.035	0.302	0.001	2.814	1.559	5.083	3.427	2.08
Endometrial thickness (>9.36 mm)	1.418	0.314	<0.001	4.130	2.232	7.643	4.516	2.73
Insemination to (1)	0.788	0.421	0.061	2.200	0.963	5.025	2.193	1.33
Constant	-4.612	0.581	0.000	0.010				
r2 = 0.191, percentage correct = 86.9%								

BMI: body mass index, TMSC: total motility sperm count, TPSC: total progressive sperm count, SE: standard of error, Exp (B): exponent of coefficient

**Determining the scoring value of each variable:** scoring values for each variable were determined based on the regression coefficient (β) and standard error obtained from the multiple logistic regression model. The updated scoring values for both male and female prognostic factors are presented in Table 4. The total prognostic factor score, based on scoring results, ranged from 0 to 9. In this study, infertile couples who became pregnant after IUI had an average total score between 5 and 6, while those who did not achieve pregnancy had an average score of around 4. Table 5 presents the results of the comparative test of total scores between pregnant and non-pregnant groups. A significant difference (p<0.001) was observed based on the comparative test of the total prognostic factor scores for post-IUI pregnancy.

**Table 4:** scoring results of post-intrauterine insemination pregnancy prognostic factors

Prognostic factors	Scoring
**Male BMI**	
<27.5 kg/m^2^	1
≥27.5 kg/m^2^	0
**Female age**	
<30.5 years	1
≥30.5 years	0
**TPSC post-preparation**	
>15.81 million	1
≤15.81 million	0
**Insemination**	
1	1
>1	0
**AMH levels**	
>3.015 ng/mL	2
≤3.015 ng/mL	0
**Endometrial thickness**	
>9.36 mm	3
≤9.36 mm	0

BMI: body mass index, TMSC: total motility sperm count, TPSC: total progressive sperm count, AMH: anti-mullerian hormone

**Table 5:** total difference test for post-intrauterine insemination pregnancy prognostic factor scores

Group	Number	Mean	P-value
Not pregnant	383	3.919	<0.001
Pregnant	60	5.867	

## Discussion

This study aims to evaluate several prognostic factors from both males and females concerning pregnancy outcomes post-IUI among infertile couples in Indonesia. Couples subjected to IUI with complete medical records were included as samples. Prognostic factors used were drawn from previous studies [5,12,14,15,20]. The factors are routinely evaluated before IUI procedures. Those significantly associated with post-IUI pregnancy and included in the scoring system of this study were divided into pre-cycle (female age, male BMI, AMH levels), insemination (TMSC and endometrial thickness), and post-insemination (TPSC post preparation and number of insemination). Others comprising factors pre-cycle, such as male age and female BMI, etiology of infertility in women; and insemination, such as sperm preparation method, type of ovarian stimulation, and the size and number of mature follicles, were not significantly associated with post-IUI pregnancy and were omitted from the scoring system.

The success rate of IUI in this study was approximately 13.5%, comparable with reported values between 8.2% and 15.1% (7-9). In the demographic analysis and frequency distribution of a sample, the average age of males and females subjected to IUI was 32.7 and 30.8 years, respectively. The age is slightly younger compared to some previous investigations [2,13,14,23]. This could be attributed to the younger average age of marriage in Indonesia [24]. Supporting the observation, Bennett *et al*. stated that the average age of females attending fertility clinics was 26 years [25].

The average BMI of females in this study was 24 kg/m^2^, similar to the general value in Indonesia, according to a 2023 study [26]. However, it is lower compared to other studies of females subjected to IUI. Studies by Soria *et al*. and Muthigi *et al*. reported an average BMI of 26 kg/m^2^ [4,12]. This difference could be due to the generally higher average BMI in European and American populations, where the investigations were conducted [27]. Supporting the observations, a 2023 meta-analysis and systematic review showed that female BMI was not significantly associated with pregnancy outcomes post-IUI [28]. Meanwhile, data on the relationship between male BMI and post-IUI pregnancy rates remains limited. Existing studies stated that obesity in males was associated with decreased sperm quality, but only a few examined the impact on post-IUI outcomes [29]. The relationship between male BMI and pregnancy is often indirect. In obese males, increased leptin levels due to higher lipogenesis in adipose cells disrupt the hypothalamic-pituitary-testicular axis, impairing testosterone production, which is crucial for spermatogenesis [30]. Additionally, oxidative stress, such as elevated levels of O_2_-, is increased in obese males, negatively affecting sperm quality [31].

Other prognostic factors that were significantly associated with post-IUI pregnancy were AMH and endometrial thickness. The mean AMH value was higher compared to a previous study by Luo *et al*. [20] Anti-Müllerian hormone (AMH) is secreted by granulosa cells within the ovarian follicles, and its levels naturally decrease with maternal age. A reduced ovarian reserve is associated with a significantly lower likelihood of achieving a clinical pregnancy (CP). While AMH is recognized as a marker of ovarian reserve in individuals undergoing fertility treatment, its levels can serve as a guide for treatment expectations and management. Numerous studies on AMH have demonstrated a correlation between AMH levels and ovarian response to stimulation [32].

Factor insemination, like endometrial thickness, has been proven to be significantly correlated to post-IUI pregnancy in other studies (5,14). Factors that had no association were sperm preparation method, type of ovarian stimulation or induction, and the size and number of mature follicles. This is consistent with the report where the relationship between the factors and post-IUI pregnancy rates varies significantly [12,13,33]. Male factor most studied about post-IUI pregnancy is the total number of motile sperm inseminated. Existing reports show that motile sperm count is significantly associated with post-IUI pregnancy [5,7,8,21]. However, the minimum threshold post-preparation capable of enhancing the probability of pregnancy post-IUI remains uncertain. Michau *et al*. stated that a TMSC of more than 5 million is associated with higher pregnancy rates [8], while in the study conducted by Gubert *et al*. the same threshold of 5 million was significant only for males under 35 years old [7]. Another study by Muthigi *et al*. recommended a higher minimum threshold of 9 million [12]. The suggested cutoff value post-preparation was 15.81 million. The lowest TPSC in couples who became pregnant post-IUI was 5.01 million.

The results showed that the first insemination is a significant prognostic factor for IUI success. However, second or subsequent inseminations in the same couple had lower success rates compared to the first. This differs from previous investigations, where the second and third inseminations had higher pregnancy rates compared to the first. Muthigi *et al*. and Soria *et al*. stated that increasing the number of IUI cycles up to the second or third significantly increased fertility [4,12]. Another study conducted by Zippl *et al*. evaluated the success rates of the administered treatment. The results showed a higher increase where the probability of pregnancy could more than triple by the sixth insemination compared to the first [21]. However, physiological factors can influence the decrease in success rates of second and subsequent cycles. IUI as an infertility therapy has considerable costs and high uncertainty, leading to increased stress for both partners. Studies have shown that stress levels increase significantly after unsuccessful infertility treatment [34]. The failure of the administered therapy can also increase the incidence of depression, which, alongside psychological stress, affects the success of subsequent cycles [23]. Differences in results in this study can be attributed to a small number of couples experiencing 3 to 4 IUI cycles. The higher pregnancy rates in the first treatment should be considered before deciding on subsequent cycles in cases of failure. It is important to recognize that pregnancy rates after the second or subsequent IUI may not increase the probability of success compared to the first. The probability may increase when other modifiable prognostic factors are significantly improved before the next cycle. The second IUI increases costs and psychological burden; hence, it should only be recommended when proven to be more effective [35].

The etiology of female infertility in this study was not related to post-IUI pregnancy rates. Furthermore, it was divided into 4, namely none or unknown, PCOS, endometriosis, and uterine disorders. Other studies analyzing the relationship between the etiology of infertility and pregnancy rates post-IUI also showed mixed results. Luo *et al*. Gubert *et al*. and Huniadi *et al*. stated that the etiology of infertility was not significantly related to post-IUI pregnancy rates [7,14,20]. Meanwhile, Michau *et al*. and Dinelli *et al*. [5,8] identified a significant relationship. The difference in results could be attributed to the use of very diverse etiological classifications of infertility. Gubert *et al*. categorized Infertility into unexplained problems in males, females, or both without specifically subdividing female infertility etiologies [7]. Huniadi *et al*. classified infertility into male, female, a combination of both, and unexplained [14]. Michau *et al*. divided the etiological factors of female into ovulation, cervical disorders, endometriosis, and low ovarian reserve [8]. Meanwhile, Dinelli *et al*. shared the same factors as Michau *et al*. without low ovarian reserve classification [5,8]. The etiology of female infertility, which was not significantly related to post-IUI pregnancies, was attributed to differences in selecting the partner to be subjected to insemination-based indications. Since each fertility clinic has highly varied protocols and clinical pathways, there is a possibility that moderate or severe etiologies of female infertility will not be inseminated. The background of female infertility conditions, which may be less diverse, causes the etiology significantly related to post-IUI pregnancy [36].

Other factors influencing clinical pregnancy outcomes are insulin resistance, LH, FSH, testosterone, and estradiol. In women, insulin resistance may affect oocyte quality by damaging oocytes through telomeres and spindle disruption, leading to embryonic development arrest, impaired endometrial tolerance, recurrent miscarriage, and inflammatory responses [37]. In men, insulin resistance can impact the concentration and production of testosterone, reduce semen volume, and decrease the percentage of progressively motile sperm [38]. The LH, FSH, testosterone, and estradiol levels in men can influence spermatozoa quality. Deoxyribonucleic acid (DNA) fragmentation analysis should be considered as a predictor of insemination outcomes, as sperm deoxyribonucleic acid (DNA) damage can lead to a reduced success rate of pregnancy [39]. However, these factors may be considered less practical as the necessary testing facilities are not always available in all healthcare centers, making it difficult to consider them in scoring factors in IUI.

**Comparison with other post-IUI pregnancy prognostic factor scoring system studies:** the outcome of this study is a prognostic factor scoring system consisting of male BMI, female age, TPSC post-preparation, AMH levels, endometrial thickness, and first insemination. Each prognostic factor is assigned a score ranging from 1 to 3, with a total score ranging from 0 to 9. Each total score corresponds to a different probability of post-IUI pregnancy. References were drawn from 2 investigations with prognostic factor scoring system outcomes in 2021 and 2022. The study by Luo *et al*. (2021) focused on prognostic factors for post-IUI pregnancy among infertile couples in China [20]. Due to comparable success rates (13.6% in Luo *et al*. study and 13.0% in our study) and equivalent statistical methods, specifically backward stepwise multivariate binary logistic regression, prognostic factors included in the final scoring system were stimulation type (natural cycle or stimulated cycle), female age, single or double IUI, as well as parameters related to sperm motility and morphology obtained through computer-assisted sperm analysis (CASA) [20]. Meanwhile, more comprehensive and specific parameters for sperm motility and morphology evaluation were provided. The prognostic factor scoring of this study was more comprehensive because it included additional factors such as male BMI, AMH levels, and endometrial thickness. The evaluated TPSC in the investigation conducted by Luo *et al* [20]. was unrelated to post-IUI pregnancy. On the contrary [7,8,12,14,33], stated that total post-preparation motile sperm count was a significant prognostic factor for pregnancy. Another comparable aspect was the use of a user-friendly and less practical scoring system. The outcome was in the form of formulas where each numerical value needed to be inputted individually and calculated manually, facilitating errors and less straightforwardness.

The latest report on prognostic factors for post-IUI pregnancy, using a comparative scoring system, is the study by Zippl *et al*. [21]. This study recorded an overall pregnancy rate of only 10.9%. Furthermore, the clinical scoring system includes factors such as female age, TPSC, female infertility etiology, and AMH levels. Other factors such as female BMI were also evaluated in this study but not included in the final scoring. However, BMI, type of stimulation, mature follicles, and endometrial thickness were not evaluated in this study. The scoring system developed is user-friendly, allowing inputs of 0, 1, or 2 for each prognostic factor. The inputs were then summed and adjusted to determine the chances of post-IUI pregnancy directly. This ease of use has led to the adoption of a similar scoring system, enhancing the applicability of the assessment. The highest success probability reported in the Australian study exceeded 60%, which is above the 55.16% reported in this study. A significant difference between the studies lies in the determination of cutoff values. For AMH, TPSC, and BMI parameters, the values used are not based on the characteristics of the sample population and are derived from studies not comprising Indonesians, hence becoming less suitable for adoption. In this study, the cutoff values are determined based on statistical tests, allowing for a better depiction of the characteristics of infertile couples.

The difference between this study and the 2 prognostic factor scoring investigations conducted by Luo *et al*. and Zippl *et al*. [20,21] lies in the parameter of TPSC used. This study evaluates post-preparation sperm parameters, while the previous 2 applied TPSC and TMSC values before preparation. Post-preparation TPSC was evaluated in prognostic factor scoring because this sperm was inseminated indirectly, hence, more specifically representing the sperm factor in males. The 2 studies also used populations in Austria and China. Furthermore, the differences in backgrounds and characteristics compared to the Indonesian population are factors that should be considered when adopting a scoring system. This presents the importance of developing prognostic factor scoring for post-IUI pregnancy with populations of infertile couples in Indonesia, as well as cutoff values and parameters that can specifically represent the population. The user-friendly and applicable output of the scoring system enables rapid and holistic use for educational purposes and decision-making. Additionally, prognostic factors from both the male and female sides should be carefully and equally considered before making decisions for IUI.

**Limitations of this study:** this study was observational with a retrospective cohort design, utilizing data from medical records. Based on this design, certain prognostic factors related to post-IUI pregnancy, which are not recorded in medical records, cannot be evaluated. Examples of these factors include difficulties encountered during the IUI procedure, DNA fragmentation index (DFI), sperm morphology, and other clinical parameters related to male infertility, such as smoking history or varicocele. Therefore, the probability of post-IUI pregnancy obtained from the scoring system and the application should be approached with caution.

## Conclusion

There is a relationship between the male partner's BMI, the total number of motile sperm after sperm preparation, the female partner's age, AMH levels, endometrial thickness, and prognostic factors from both the male and female sides with pregnancy after IUI. There is no relationship between the male partner's age, sperm preparation method, female partner's BMI, female infertility etiology, type of stimulation, and the size of the mature follicle post-stimulation with pregnancy after IUI. We suggest that in daily practice, significant variables such as male BMI, female age, TPSC, AMH levels, and endometrial thickness are factors that need to be corrected before IUI to support therapeutic success. The path analysis approach is one method that can be recommended for future research to assess the relationship of each variable in supporting the success of IUI.

### What is known about this topic

- Intrauterine Insemination (IUI) was selected as a treatment for infertility due to the simple method, affordability, and non-invasive procedure other than IVF procedure;
- Success rate IUI considerably raised if this couple had minimal etiology factors for their infertility;
- Lack of information about pregnancy programs for couples affects the decreased success rate of pregnancy programs.

### What this study adds

- This study reports the scoring in the Indonesian population by including two variables, total progressive sperm count (TPSC) and endometrial thickness, which were not previously reported in other studies;
- Our study showed different results, such as the first insemination attempt having a greater pregnancy chance than the second or third attempt.
